# Supplementary material for: Empirical methods for controlling false positives and estimating confidence in ChIP-Seq peaks
Source: BMC Bioinformatics. 2008 Dec 5;9:523. doi: 10.1186/1471-2105-9-523 (PMC2628906; doi:10.1186/1471-2105-9-523)
Supplement: Additional File 1 — A variety of html documents from the USeq web site detailing the available applications, their best usage, output file type descriptions, command line menus, etc. [file 1471-2105-9-523-S1.zip › USeqUserGuides/JavaDoc.zip!/JavaDoc/trans/roc/Gr.html]

Gr


|  |  |  |
| --- | --- | --- |
| **Overview** \| **Package** \| **Class** \| **Use** \| **Tree** \| **Deprecated** \| **Index** \| **Help** | |  |
| **PREV CLASS**   **NEXT CLASS** | **FRAMES**    **NO FRAMES**     **All Classes** |  |
| SUMMARY: NESTED \| FIELD \| CONSTR \| METHOD | DETAIL: FIELD \| CONSTR \| METHOD |  |


---


## trans.roc Class Gr

```
java.lang.Object
  trans.roc.Gr
```

---

``` public class Gr extends java.lang.Object ```

Gr line container.

---

**Constructor Summary**

`Gr(int position,
float score)`

`Gr(java.lang.String line)`


| **Method Summary** | |
| --- | --- |
| `int` | `getPosition()` |
| `float` | `getScore()` |
| `static Gr[]` | `parseGrGraph(GrGraph gr)` |
| `void` | `setPosition(int position)` |
| `void` | `setScore(float score)` |
| `java.lang.String` | `toString()` |

**Methods inherited from class java.lang.Object**

`equals, getClass, hashCode, notify, notifyAll, wait, wait, wait`

**Constructor Detail**

### Gr

```
public Gr(java.lang.String line)
```

---


### Gr

```
public Gr(int position,
          float score)
```


**Method Detail**

### parseGrGraph

```
public static Gr[] parseGrGraph(GrGraph gr)
```

---


### getPosition

```
public int getPosition()
```

---


### getScore

```
public float getScore()
```

---


### toString

```
public java.lang.String toString()
```

:   **Overrides:**: `toString` in class `java.lang.Object`

---


### setScore

```
public void setScore(float score)
```

---


### setPosition

```
public void setPosition(int position)
```


---


|  |  |  |
| --- | --- | --- |
| **Overview** \| **Package** \| **Class** \| **Use** \| **Tree** \| **Deprecated** \| **Index** \| **Help** | |  |
| **PREV CLASS**   **NEXT CLASS** | **FRAMES**    **NO FRAMES**     **All Classes** |  |
| SUMMARY: NESTED \| FIELD \| CONSTR \| METHOD | DETAIL: FIELD \| CONSTR \| METHOD |  |


---
